# Hormonal therapy is effective and safe for cryptorchidism caused by idiopathic hypogonadotropic hypogonadism in adult males

**DOI:** 10.3389/fendo.2022.1095950

**Published:** 2023-01-18

**Authors:** Taotao Sun, Wenchao Xu, Hao Xu, Yinwei Chen, Yonghua Niu, Daoqi Wang, Tao Wang, Weimin Yang, Jihong Liu

**Affiliations:** ^1^ Department of Urology, Tongji Hospital, Tongji Medical College, Huazhong University of Science and Technology, Wuhan, China; ^2^ Institute of Urology, Tongji Hospital, Tongji Medical College, Huazhong University of Science and Technology, Wuhan, China; ^3^ Reproductive Medicine Center, Tongji Hospital, Tongji Medical College, Huazhong University of Science and Technology, Wuhan, China; ^4^ Department of Pediatric Surgery, Tongji Hospital, Tongji Medical College, Huazhong University of Science and Technology, Wuhan, China; ^5^ Department of Urology, The Second Affiliated Hospital of Kunming Medical University, Kunming, China

**Keywords:** cryptorchidism, idiopathic hypogonadotropic hypogonadism, hormonal therapy, testicular descent, spermatogenesis

## Abstract

**Background:**

Hormonal therapy is a reasonable treatment for cryptorchidism caused by idiopathic hypogonadotropic hypogonadism (IHH). However, the clinical evidence on whether it is effective and safe for the treatment of cryptorchidism caused by IHH is lacking.

**Aim:**

To evaluate the effect of hormonal therapy in testicular descent, puberty development, and spermatogenesis in adult males with cryptorchidism caused by IHH.

**Methods:**

This retrospective study included 51 patients with cryptorchidism caused by IHH from the Andrology Clinic of University affiliated teaching hospital. Patients were divided into two groups: group A patients received hormonal therapy; group B patients received surgical treatment for cryptorchidism followed by hormonal therapy.

**Results:**

The rate of successful testicular descent following hormonal therapy (19/32 in group A) or surgical treatment (11/19 in group B) shows no statistically significant difference. There was also no statistically significant difference in penile length, Tanner stage of pubic hair, testicular volume, and success rate of spermatogenesis between the two groups. Testicular atrophy was seen in a single patient in group B.

**Conclusions:**

Hormone therapy in adult males with cryptorchidism caused by IHH is effective and safe regarding testicular descent, puberty development, and spermatogenesis. This study provides new insight into the treatment of cryptorchidism caused by IHH and highlights that hormonal therapy could be an effective, safe, and economic treatment option for cryptorchidism in males caused by IHH.

## Introduction

Cryptorchidism or undescended testes is the failure of the testis to descend into its normal scrotal position; it may be unilateral or bilateral (1). The incidence of cryptorchidism varies and depends on gestational age (2). At 15 years of age, the incidence varied from 1.6 to 2.2%, whereas at an older age, cryptorchidism became rare (3). Idiopathic hypogonadotropic hypogonadism (IHH) is a rare but treatable male infertility disorder caused by a congenital defect in the gonadotropin-releasing hormone (GnRH), including deficient development and migration of GnRH neurons or deficient GnRH secretion and action (4, 5). Anosmia or hyposmia in the presence of IHH is classed as Kallmann syndrome (KS), while IHH patients with a normal sense of smell are defined as normosmic IHH (nIHH) (1). Because testicular descent requires normal hypothalamo-pituitary-gonadal axis function and androgen secretion and action, IHH is one of the syndromes causing cryptorchidism (1, 3). Due to the lack of large-sample studies, the exact prevalence of cryptorchidism in IHH patients is unknown. However, in some studies with small samples, the prevalence of cryptorchidism in IHH patients varies from 38% to 69.6% (1), which is much higher than that in the general population.

Cryptorchidism is a clinical condition that increases the risks of infertility, testicular malignancy, and inguinal hernia and needs proper treatment (6). In the past decades, orchidopexy and hormonal therapy with gonadotropin or GnRH were recommended as treatment options for cryptorchidism in children (7). However, the reported studies on hormone therapy for cryptorchidism had unknown etiologies in the study population and its overall success rate was only 20% (8). So, hormonal therapy was not recommended by almost all guidelines for patients with cryptorchidism (2, 6, 8).

It has been reported that the increased testosterone in middle pregnancy and early after birth (mini-puberty) controls the differentiation of internal genitalia and testicular descent in males (1). Cryptorchidism caused by IHH is mainly the result of low testosterone levels due to the lack of GnRH and gonadotropin in these periods (1). Furthermore, the cryptorchidism caused by IHH persists in adulthood given that testosterone levels remain low in adults with IHH (5). So, hormonal therapy with gonadotropin or GnRH is a reasonable treatment for cryptorchidism caused by IHH (1). IHH infants could be diagnosed early during mini-puberty by detecting low serum FSH, LH, testosterone, and inhibin B compared to the reference ranges at 4–8 weeks after birth (5). Two small studies recently demonstrated that most male infants with IHH-associated cryptorchidism could achieve complete testicular descent after gonadotropin administration (9, 10). However, as far as we know, the study regarding hormonal therapy in testicular descent in adult males with cryptorchidism caused by IHH is completely lacking, and the efficacy and long-term outcome of hormonal therapy are unknown.

In males with IHH, the goals of treatment are to induce and maintain normal puberty and to restore fertility (1). For IHH males with cryptorchidism, achieving testicular descent is also the principal issue. Hormonal therapy has been proven safe and effective in inducing and maintaining normal puberty and restoring fertility in IHH patients (4). However, the safety and efficacy of hormonal therapy for cryptorchidism in adult IHH patients are not well evaluated. Therefore, we conducted a retrospective study to investigate the efficacy and safety of hormonal therapy in testicular descent, puberty development, and spermatogenesis in adult patients with cryptorchidism caused by IHH.

## Patients and materials

This study was approved by the Ethics Committee of Tongji Hospital, Tongji Medical College, Huazhong University of Science and Technology (TJ-IRB20150302). This retrospective study enlisted patients with cryptorchidism caused by IHH who were seen in the Andrology clinic of Tongji Hospital during the years 2008-2022. We excluded patients with incomplete medical records and without follow-up data. The diagnosis of IHH was based on the reported criteria, as follows: 1) incomplete or absent puberty at the age of 18 years; 2) low serum testosterone level and low or normal levels of gonadotropins; 3) otherwise normal pituitary function; 4) normal hypothalamic-pituitary imaging findings (4, 11). Notably, three of the patients under the age of 18 years were also diagnosed with KS because of anosmia; absent puberty; low serum testosterone, LH, and FSH levels; otherwise normal pituitary function; and normal hypothalamic-pituitary imaging findings (11). For the other seven patients who were under the age of 18 years and osmatic but in line with the clinical features of IHH, the hormonal therapy was suspended for six months when they were 18 years old to successfully distinguish IHH from the constitutional delay of growth and puberty (CDGP). The diagnosis of cryptorchidism was based on history, physical examination, and ultrasonography.

According to their treatment history, patients were divided into two groups: group A patients received hormonal treatment, and group B patients were treated with orchidopexy followed by hormonal treatment. Orchidopexy was performed *via* an inguinal approach. Hormonal treatment was administered in the following regime. When treated with gonadotropin, 2000 IU of hCG was injected intramuscularly twice per week in the first three months. After a minimum of six months of hCG treatment, 75~150 IU of hMG was added *via* intramuscular injection twice a week to further enhance spermatogenesis. When treated with pulsatile GnRH, 10μg of GnRH (200 μg/mL) was subcutaneously injected every 90 minutes with a pump in the initial three months. The dose of hCG and GnRH was increased if the testosterone level was below the normal reference range.

Height and body weight, location of the testis, testicular volume, sex hormones, penile length, Tanner stage of pubic hair, and semen analysis were evaluated and recorded at the first visit and every 3-6 months thereafter. Testicular volume was measured by ultrasonography and the volume was calculated using the formula π/6×length×height×width or Prader orchidometer (12). The stretched penile length was measured from the root of the penis at the pubic symphysis to the top of the glans in the flaccid states (13). The Tanner stage of pubic hair was evaluated by a single physician according to the Tanner staging (14). Sex hormones including serum testosterone, LH, and FSH were measured with chemiluminescent immunoassay every 3-6 months. Early morning blood samples were collected for the measurement of sex hormones. Semen analysis was performed in patients who were able to produce an ejaculate. Semen volume, sperm concentration, and sperm motility were examined, according to the WHO Laboratory Manual for the Examination and Processing of Human Semen (fifth version). The location of the testes was assessed by physical examination and ultrasonography of the scrotum and groin and described as abdominal, inguinal, supra-scrotal, high scrotal, and scrotal (normal) according to the criteria in a previous report (1). Postoperative complications including incision infection and testicular atrophy and potential side effects including severe acne, gynecomastia, frequent erection, pain during erection, injection site infection or pain, allergic skin reaction, testicular tumor, and other discomfort associated with hormonal therapy were also analyzed.

SPSS Statistics 24.0 software was used for statistical analysis. Continuous data were analyzed using the median and interquartile range and compared by the Student t-test. Categorical variables were compared using the chi-square test or Fisher exact test. P<0.05 was considered statistically significant.

## Result

### Baseline characteristics

**Figure 1:**
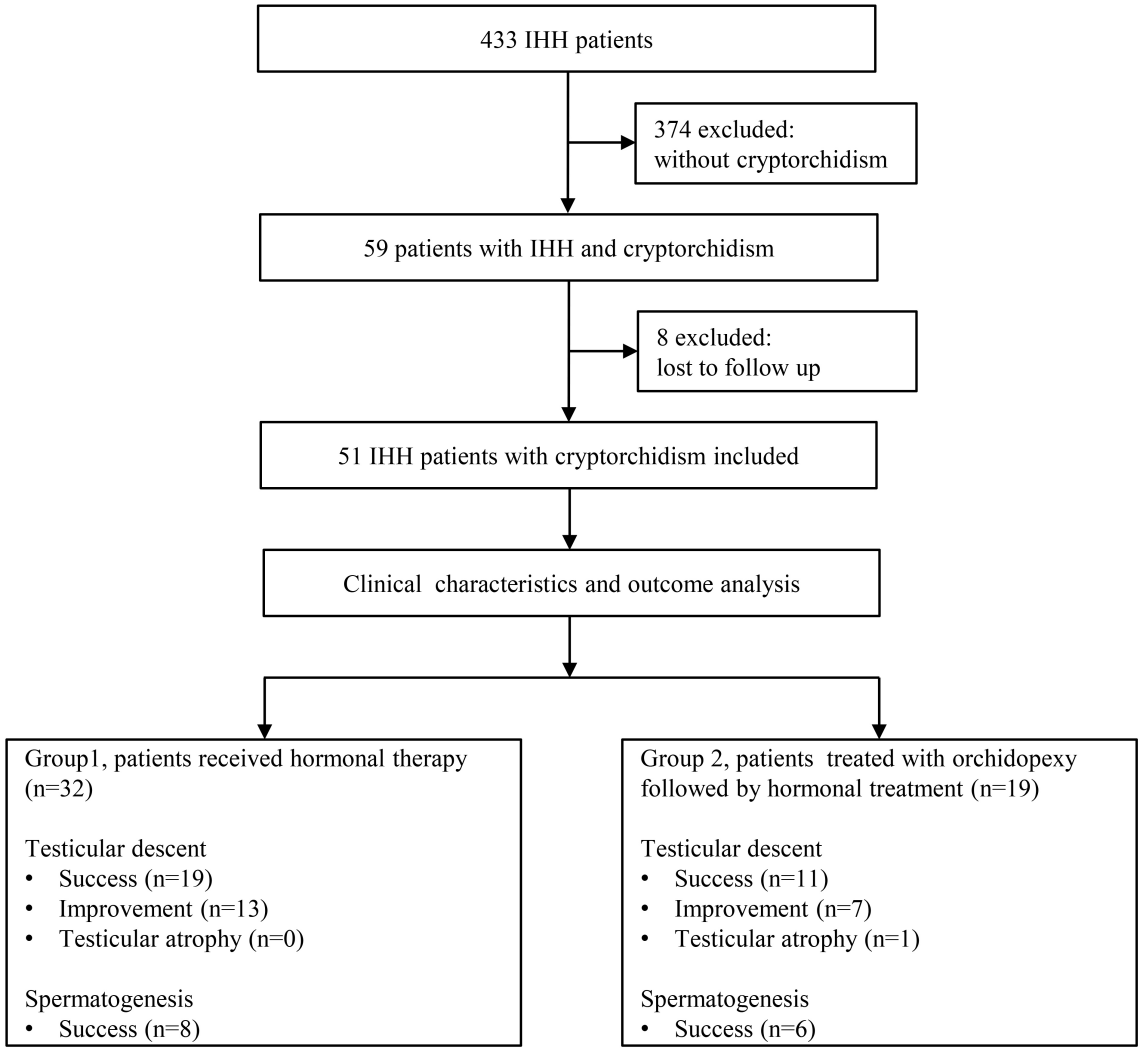
Study flow diagram. IHH, Isolated hypogonadotropic hypogonadism.

We identified 59 patients with cryptorchidism in 433 IHH patients, which makes the prevalence of cryptorchidism in IHH patients in our cohort 13.6%. Finally, a total of 51 patients with IHH and cryptorchidism were identified for inclusion in this study (**Figure 1**). Patients’ characteristics before hormonal therapy are shown in **Table 1**. Of the 51 patients, 32 patients (16 KS patients and 16 nIHH patients) received hormonal treatment (group A), and 19 patients (14 KS patients and 5 nIHH patients) were treated with orchidopexy followed by hormonal treatment (group B). In group A, 28 patients were treated with hCG combated with hMG, one was treated with hCG combated with hMG followed by pulsatile GnRH pump, and three were treated with a pulsatile GnRH pump only. In group B, 17 patients were treated with hCG combined with hMG, one was treated with hCG combined with hMG followed by pulsatile GnRH pump, and another was treated with a pulsatile GnRH pump only after orchidopexy.

**Table 1:** Basic characteristics of the IHH patients with cryptorchidism in the two groups.

	Group A (n=32)	Group B (n=19)	*P value*
Age, years	21.0 (18.0,27,0)	20.0 (18.0,22.0)	0.194
<20	10	8	–
20-30	18	10	–
>30	3	1	–
Height, cm	170.0 (164.3,175.0)	169.5 (160.5,177.3)	0.856
Weight, kg	61.0 (54.8, 80.6)	58.5 (55.0,71.5)	0.575
BMI, kg/m^2^	22.2 (19.1, 27.8)	21.5 (19.8, 24.4)	0.451
Diagnosis			0.097
KS	16	14	–
nIHH	16	5	–
Cryptorchidism			0.539
Bilateral	18	9	–
Unilateral	14	10	–
Tanner stage of pubic hair	1 (1,2)	1 (1,2)	0.594
Penis length, cm	3.0 (2.3,3.8)	3.0 (3.0,4.0)	0.663
Testicular volume, ml	1.5 (1.0,4.0)	2.0 (1.0,3.3)	0.893
FSH, mIU/ml	0.9 (0.5,1.2)	1.0 (0.5,1.4)	0.565
LH, mIU/ml	0.4 (0.2,0.6)	0.2 (0.2,0.4)	0.006
Total testosterone, ng/ml	0.3 (0.1,0.4)	0.4 (0.2,0.4)	0.083

BMI, body mass index; KS, kallmann syndrome; nIHH, normosmic idiopathic hypogonadotropic hypogonadism.

All the participants in both groups had unilateral or bilateral undescended testes, which were located below the Inner ring of the inguinal canal. The detailed positions of testes in patients pre- and post-treatment are shown in **Supplementary table 1**. There were no significant differences in age, height, body weight, testicular volume, percentage of bilateral cryptorchidism, and testicular position between the two groups at the beginning of the hormonal therapy.

### Comparison of the effects of hormonal therapy and surgical therapy for cryptorchidism

**Table 2** presents the effects of hormonal therapy and surgical therapy for IHH-related cryptorchidism in the two groups. In group A, 11 of the 18 patients with bilateral cryptorchidism had both testes descended into the normal position in the scrotum after the hormonal therapy. Among the remaining patients, two patients only had a single testis migrating into the normal position in the scrotum, while the other five had bilateral testes descending further distally in the upper scrotum position. Among the 14 unilateral cryptorchidism patients, eight had their testis descended into the normal position in the scrotum, one descended to a high scrotal position, and the other five only progressed more distally in the upper scrotum position. The success rate, measured as complete testicular descent into the normal position in the scrotum after hormonal therapy for both bilateral and unilateral cryptorchidism, was 59.4% (19/32). The improvement rate, measured as progress in the position of the testes in patients with unilateral cryptorchidism, one of their two testes migrating into the normal position in the scrotum, or progress in the position of the testes in patients with bilateral cryptorchidism, was 40.6% (13/32). The median time it took for the testes to descend into the scrotum was 10.0 (6.0, 19.0) months after hormonal therapy.

**Table 2:** The effect of hormonal therapy and surgery for cryptorchidism in IHH patients.

Cryptorchidism	Group A (n=32)	Group B (n=19)	*P value*
Success	19	11	0.450
bilateral	11	4	–
unilateral	8	7	–
Improvement	13	7	1.000
bilateral	7	4	–
unilateral	6	3	–
Testicular atrophy	0	1	–
bilateral	0	1	–
unilateral	0	0	–

In group B, bilateral testes were successfully placed in the normal position in the scrotum after orchidopexy in four patients (4/9). In two patients with bilateral incompletely descended testes, one testis was in the normal position in the scrotum following surgery, but the other testis could not be mobilized from the upper scrotum position. However, the undescended testis descended into the normal position in the scrotum after five months following the hormonal treatment course. Another two patients with bilateral cryptorchidism both underwent surgery twice to mainly mobilize the right testis to the normal position in the scrotum. However, the right testis did not descend into the scrotum after the surgery and could not descend into the scrotum during the hormonal treatment. Unfortunately, bilateral testicular atrophy was observed in one patient with bilateral cryptorchidism, which was corrected with orchidopexy. Seven patients who had unilateral cryptorchidism had their testes brought into the normal position in the scrotum and one testis could only be mobilized to the high scrotal position in the other two patients. The remaining one other only progressed more distally in the upper scrotum position. Overall, in group B, the success rate of orchidopexy for both bilateral and unilateral cryptorchidism was 57.9% (11/19), the improvement rate was 36.8% (7/19) while the testicular atrophy rate was 5.3% (1/19). There was no statistical difference in the success rate of hormonal therapy and surgical treatment for cryptorchidism in the two groups (p=0.917).

Notably, two patients in group A eventually converted to sliding cryptorchidism. In one patient, the left testis descended into the normal position in the scrotum after ten months of hormone therapy. The other patient had bilateral cryptorchidism, and the testes descended into the scrotum after 50 months of hormone therapy. However, both patients had been off medication for five months at the follow-up and during this period, their testes had retracted to a high scrotal position. Fortunately, the testes descended again after the treatment resumed in both patients.

Considering that the severity of cryptorchidism, genetics (gene mutations associated with IHH) (15), and treatment methods differed among different patients, we analyzed the influence of the above factors on testicular descent. The results show that no difference was observed based on the severity of cryptorchidism (**Supplementary table 2**), genetics (**Supplementary table 3**), and treatment given (**Supplementary table 4**).

### Extended follow-up results for incomplete testicular descent in group A

Among the 13 patients with IHH-related cryptorchidism whose testis didn’t descend into the normal position in the scrotum temporarily in group A, ten of them chose orchidopexy to achieve better therapeutic results after the median time of 12 months following hormonal therapy. All of them had testes descended into the normal position in the scrotum regardless of bilateral or unilateral cryptorchidism except two patients. One received orchidopexy for bilateral cryptorchidism after the initial 21 months of hormonal therapy then went through 13 months of hormonal therapy again. As a result, the left testis was in the normal position in the scrotum while the right was in a high scrotal position. The other received hormonal therapy for 12 months followed by orchidopexy for the right cryptorchidism. This resulted in a high scrotal position after 76 months of follow-up hormone therapy.

### Comparison of the effects of hormonal therapy in inducing puberty and restoring fertility

**Table 3** presents the effects of hormonal therapy in inducing puberty and restoring fertility between the two groups. After the hormonal therapy, the mean testosterone levels, Tanner stage of pubic hair, testicular volume, and penile length significantly improved in both groups. However, there was no statistical difference in the mean duration of follow-up, mean testosterone levels, FSH, LH, Tanner stage of pubic hair, testicular volume, and penis length between the two groups.

**Table 3:** The effects of hormonal therapy in patients in the two groups.

	Group A (n=32)	Group B (n=19)	P value
Follow-up period, months	39.0 (19.3, 58.5)	38.0 (27.0,63.0)	0.792
Tanner stage of pubic hair	5 (3,5)	5 (4,5)	0.604
Penis size, cm	6.0 (5.0,6.1)	5.0 (4.0,6.0)	0.197
Testicular volume, ml	8.0 (6.0,11.0)	6.5 (5.0,10.3)	0.522
FSH, mIU/ml	1.0 (0.3,2.3)	1.4 (0.5,3.0)	0.691
LH, mIU/ml	0.2 (0.2,0.5)	0.3 (0.2,0.6)	0.894
Total testosterone, ng/ml	2.3 (1.2,3.6)	1.8 (1.3,2.1)	0.092
Nocturnal emission	32	19	–
Semen analysis			
Involved patients	16	7	–
Mean semen volume, ml	1.70 ± 0.90	1.89 ± 2.39	0.786
Spermatozoa appearance	8	6	0.611
Sperm concentration, ×10^6^/ml	8.40 ± 8.03	65.14 ± 82.70	0.154
Percentage of a+b sperm, %	20.98 ± 12.69	22.85 ± 15.89	0.811

The above indicators were all detected under treatment.

Nocturnal emission occurred in all patients during treatment in both groups. In group A, two patients impregnated their partners after receiving hormonal therapy for 15 months and 73 months respectively. In group B, three patients impregnated their partners after receiving hormonal treatment. There was no statistical difference in the success rate of spermatogenesis (p=0.611), the mean semen volume (p=0.786), the sperm concentration (p=0.154), and the percentage of grade a and b sperm (p=0.811) in the two groups.

### Safety assessment results

During the hormonal treatment, complications like frequent erection, pain during erection, and injection site infection or pain were not reported in either group. One patient in group A exhibited a skin rash on the chest and back and another patient in group B exhibited breast enlargement during the hormonal treatment. No testicular tumor was detected in either group during follow-up.

## Discussion

Low levels of testosterone since the fetal stage result in a high incidence rate of undescended testis in IHH males (14). In our study, we investigated the incidence rate of cryptorchidism in a relatively large cohort of IHH for the first time. Because of the sample size, we believe this is the most accurate report regarding the incidence rate of cryptorchidism in IHH patients to date.

It has been proven that hormonal therapy, which could increase the serum T level in IHH patients, is a reasonable treatment for cryptorchidism caused by IHH, and its efficiency has been reported in infants with IHH-related cryptorchidism (1, 9, 10). In our study, we investigated the effect of hormone therapy on adult patients with cryptorchidism caused by IHH. We found that more than half of the adult IHH males with cryptorchidism achieved complete testicular descent after hormonal therapy. There was no statistical difference in the success rate of hormonal therapy and surgery for cryptorchidism in IHH patients. Unfortunately, bilateral testicular atrophy, the most serious complication associated with surgical treatment (16), was found in one of the patients who received surgery for the treatment of bilateral cryptorchidism. No testicular atrophy was found in patients who received only hormonal treatment. Thirteen patients in group A failed to have the testis descend to the bottom of the scrotum during the hormonal treatment. However, the location and volume of the testes showed visible improvement after hormonal treatment in all of them, which facilitated the following orchidopexy. In fact, 80% (8/10) of them achieved satisfactory testicular descent during the subsequent surgery. Our results suggest that hormonal therapy is effective in testicular descent, avoids surgical challenges and complications in most cases of cryptorchidism in adult males caused by IHH, and facilitates the subsequent orchidopexy in the rest of them. Besides, although many studies have demonstrated the importance of testosterone in male testicular descent during middle pregnancy and early after birth (1, 3), our results also confirm this role in adult males.

Inducing puberty and restoring fertility are two major treatment goals in IHH males (17). Our data shows that hormonal therapy could induce puberty effectively in IHH males with cryptorchidism and is not influenced by surgical treatment. Though it has been reported that cryptorchidism is a negative prognostic factor for spermatogenesis in men with IHH (18, 19), some of our patients produced sperm successfully after hormonal therapy. There was no statistical difference in the success rate of spermatogenesis after hormonal therapy in either group. These results suggest hormonal therapy could effectively restore fertility in men with IHH and cryptorchidism and is not dependent on surgical correction for the undescended testis before hormonal therapy.

The most serious concern of cryptorchidism is testicular tumors; however, no testicular tumor was detected in our patients during the treatment. That may be due to a relatively short follow-up time and a small sample size. Another possibility is that all of the undescended testes were located outside the abdominal cavity and most of the undescended testes descended into the scrotum in a short time (median time of 10.0 months in the hormonal therapy group) after the treatment, and this may have decreased the risk of the occurrence of testicular tumors in these cryptorchidism patients. In order to minimize the risk of testicular cancer and the negative effects on spermatogenesis, we recommend orchidopexy for IHH patients with cryptorchidism who failed to achieve complete testicular descent after hormonal therapy. However, the risk of testicular cancer must be investigated in a larger number of patients with longer follow-up periods.

IHH is a rare condition and adult males with IHH and cryptorchidism are even more rare. Therefore, it is difficult to collect a large number of adult patients with cryptorchidism caused by IHH. To our knowledge, this is the first study with a relatively large sample size and long duration of follow-up time attempting to investigate the effect of hormonal therapy on testicular descent in adult males with cryptorchidism caused by IHH, which may rewrite the guideline statement on males with cryptorchidism caused by IHH. However, this study possesses a few limitations. Firstly, the sample size of this study is still not large enough, which limits the credibility of this study. For example, semen parameters with a smaller sample size did not seem to be able to produce confident conclusions. Secondly, this was a retrospective study without rigorous experimental design. Thirdly, not all indicators or hormones involved in testicular descent were included in this study, such as insulin-like peptide 3 (20). Therefore, a well-designed prospective study with an adequate sample size and more detailed clinical data should be considered in the future.

## Conclusions

In this retrospective pilot study, we demonstrate that hormone therapy in adult males with cryptorchidism caused by IHH is effective and safe in regard to testicular descent, puberty development, and spermatogenesis. This study provides new insights into the treatment of cryptorchidism caused by IHH and highlights that hormonal therapy could be an effective, safe, and economic treatment option for cryptorchidism in males caused by IHH.

## Data availability statement

The original contributions presented in the study are included in the article/**Supplementary Material**. Further inquiries can be directed to the corresponding authors.

## Ethics statement

The studies involving human participants were reviewed and approved by the Ethics Committee of Tongji Hospital, Tongji Medical College, Huazhong University of Science and Technology. Written informed consent to participate in this study was provided by the participant or their legal guardian.

## Author contributions

Conceptualization: JL, HX. Methodology: TS, WX, HX. Investigation: TS, HX, YC, YN, DW. Data curation: TS, HX, YC, YN, DW. Formal analysis: TS, WX, HX. Visualization: TS, HX. Funding acquisition: JL, HX. Supervision: TW, WY. Writing – original draft: TS, HX. Writing – review and editing: JL, WX. All authors have made substantial contributions to the study and have read and approved the final manuscript. All authors contributed to the article and approved the submitted version.
