# Supplementary material for: Hormonal therapy is effective and safe for cryptorchidism caused by idiopathic hypogonadotropic hypogonadism in adult males
Source: Front Endocrinol (Lausanne). 2023 Jan 18;13:1095950. doi: 10.3389/fendo.2022.1095950 (PMC9889536; doi:10.3389/fendo.2022.1095950)
Supplement: Supplementary file 1 [file Table_1.docx]

Supplementary Material

# Supplementary Table

|  | Pre-treatment | | | Post-treatment | | |
| --- | --- | --- | --- | --- | --- | --- |
|  | Group A  (n=50) | Group B  (n=28) | *P value* | Group A (n=50) | Group B  (n=28) | *P value* |
| Scrotal | 0 | 0 | 0.2234 | 32 | 17 | 0.2346 |
| High scrotal | 4 | 2 |  | 1 | 4 |  |
| Suprascrotal | 10 | 6 |  | 11 | 5 |  |
| Inguinal | 36 | 20 |  | 6 | 2 |  |

**Supplementary table 1.** The position of undescended testes pre- and post-treatment.

n indicates the number of testes including bilateral cryptorchidism (denoted as 2) and unilateral cryptorchidism (denoted as 1)

**Supplementary table 2.** The effects of the severity of cryptorchidism in patients of the two groups.

|  | Success | Improvement | Testicular atrophy | *P* |
| --- | --- | --- | --- | --- |
| Group A (n=50) |  |  |  | 0.258 |
| Scrotal | 0 | 0 | 0 |  |
| High scrotal | 4 | 0 | 0 |  |
| Suprascrotal | 6 | 4 | 0 |  |
| Inguinal | 20 | 16 | 0 |  |
| Group B (n=28) |  |  |  | 0.338 |
| Scrotal | 0 | 0 | 0 |  |
| High scrotal | 0 | 2 | 0 |  |
| Suprascrotal | 4 | 2 | 0 |  |
| Inguinal | 13 | 5 | 2 |  |

n indicates the number of testes including bilateral cryptorchidism (denoted as 2) and unilateral cryptorchidism (denoted as 1)

**Supplementary table 3.** The effects of different gene mutations in patients of the two groups.

|  | Mutations | No mutations | *P* |
| --- | --- | --- | --- |
| Group A (n=11) |  |  | 1.000 |
| Success | 5 | 4 |  |
| Improvement | 1 | 1 |  |
| Group B (n=11) |  |  | 1.000 |
| Success | 4 | 5 |  |
| Improvement | 1 | 0 |  |
| Testicular atrophy | 0 | 1 |  |

n indicates the number of patients with genetic testing data associated with IHH.

**Supplementary table 4.** The effects of different therapeutic strategies in patients of the two groups.

|  | Success | Improvement | Testicular atrophy | *P* |
| --- | --- | --- | --- | --- |
| Group A (n=32) |  |  |  | 0.737 |
| pulsatile GnRH | 1 | 2 | 0 |  |
| Gonadotropin | 17 | 11 | 0 |  |
| Gonadotropin  followed by GnRH | 1 | 0 | 0 |  |
| Group B (n=19) |  |  |  | 0.665 |
| pulsatile GnRH | 0 | 1 | 0 |  |
| Gonadotropin | 10 | 6 | 1 |  |
| Gonadotropin  followed by GnRH | 1 | 0 | 0 |  |
